# Routine Histopathology of Tonsillectomy Specimens, Is It Necessary? A Prospective Observational Study

**DOI:** 10.1007/s12070-024-04888-1

**Published:** 2024-07-17

**Authors:** Vidula Mestry, Ravindra B. Sardesai, Sanjaykumar Sonawale, Mihir Vaidya, Samir V. Joshi

**Affiliations:** 1 Division of Head and Neck Surgery, Department of Surgical Oncology, Tata Memorial Centre, ACTREC, Mumbai, India; 2 Department of ENT, Jehangir Hospital, Pune, India; 3 Department of ENT, B. J. Government Medical College and Sassoon General Hospital, Pune, India; 4 Department of Histopathology, Sahyadri Hospital, Pune, India; 5 K. J. Somaiya Medical College and Research Centre, Mumbai, India

**Keywords:** Chronic tonsillitis, Tonsillectomy, Histopathology, Non-Hodgkin’s lymphoma, Choristoma, Chronic granuloma, Squamous cell carcinoma

## Abstract

Routine histopathology of tonsillectomy specimens is not standard practice due to cost and resource constraints. This study aimed to evaluate the prevalence of abnormal histopathologies in tonsillectomy specimens to determine the necessity of routine histopathology. A prospective observational study was conducted from January 2014 to August 2016 at a general tertiary care center after approval of Institutional Ethics Committee (IEC). Patients scheduled for tonsillectomy surgery as per the AAOHNS 2011 criteria were included in the study. All tonsillectomy specimens were sent for histopathology, and abnormal findings were documented. Statistical analysis was performed. A total of 111 patients were enrolled in this study, and 222 tonsillectomy specimens were analyzed. The most common finding was chronic tonsillitis, which justified the selection criteria in 91.89% of patients. Two patients with chronic tonsillitis were also found to have actinomycosis present on the surface without parenchymal tissue reaction. Abnormal histopathological findings were observed in 9 (8.10%) of patients, leading to further investigations or treatment in 8 cases. These abnormalities included chronic granuloma, non-Hodgkin's lymphoma, early squamous cell carcinoma, and one choristoma. Tonsillar asymmetry, recent onset of symptoms and older age were found to be significantly correlated with abnormal histopathology and malignancy. Routine histopathology in tonsillectomy specimens helps to identify important findings that may require additional treatment. Based on the study we strongly recommend routine histopathology of tonsillectomy specimen. When significant cost constraints exist, risk-based approach can be adopted. Factors such as older age, asymmetry of tonsils, referred otalgia, duration of symptoms (recent onset) and a history of addiction should be considered for proceeding with histopathology.

**Supplementary Information:**

The online version contains supplementary material available at 10.1007/s12070-024-04888-1.

## Introduction

Tonsillectomy, the surgical removal of tonsils, is widely performed surgery across the globe, particularly in paediatric age group [1, 2]. Tonsillectomy can be therapeutic, diagnostic or as a preliminary step for another surgery [1].

The conventional practice has always deemed it necessary to perform histopathological analysis on tonsillectomy samples in order to accurately diagnose underlying conditions, such as long-standing inflammation or cancer [3, 4].

Nevertheless, there are opposing perspectives on the necessity of routine histopathological examination (HPE) for tonsillectomy specimens. Varied research findings and opinions contribute to the ongoing debate. Some studies assert that routine examination is vital as it aids in the detection of clinically significant conditions like hidden malignancies. However, contrasting evidence suggests that routine examination often yields few significant diagnoses, making it an expensive and resource-intensive practice. Patients also bear the brunt of additional costs and potential delays associated with undergoing routine histopathological examination. Moreover, this practice contributes to the overall healthcare expenditure. Another concern is the increased workload on pathologists, potentially leading to backlogs and delays in reporting urgent results.

This study is aimed to prospectively evaluate prevalence of significant histopathologies detected on routine tonsillectomy specimens and to contribute to the ongoing debate on the necessity of this practice and provide valuable insights for future decision-making.

## Methods

A prospective study was conducted in a general government tertiary care hospital following approval from the institutional ethical committee (Ref No BJMC/IEC/Pharmac/D-1213193-193 date 24/12/2013). Between January 2014 and January 2016, consecutive patients scheduled for tonsillectomy who were willing to participate in the study were included in the study, and written informed consent was obtained from patients or their guardians if they were minors. Patients not willing to participate, those with bleeding disorders, tonsillectomy performed as emergency procedure due to peritonsillar abscess were excluded from the study. Patient selection for tonsillectomy followed the guidelines outlined by the American Academy of Otolaryngology-Head and Neck Surgery (AAOHNS) in 2011 [5]. For statistical analysis, patients were divided into three age groups: Group A (5–18 years), Group B (19–40 years), and Group C (> 40 years). Demographic and clinical data were collected prospectively. Tonsillectomy (extracapsular) was performed using the standard cold steel method. Study was performed in accordance with declaration of Helsinki, 2013 amendment.

### Histopathological Examination

The resected tonsils were placed in a wide mouth container containing 10% formalin and subsequently sent to the histopathology department for histopathological examination (HPE). Following standard guidelines, the tissue processing was done. Routine hematoxylin and eosin staining was performed, and additional special staining and immunohistochemistry were employed when necessary.

### Statistical Analysis

The quantitative data collected in this study was analysed using frequencies and measures of central tendencies. Statistical significance was assessed using 2-sided Chi-squared and Fisher Exact tests. All statistical analysis was performed using SPSS version 25 (IBM, New York, USA). To ensure accurate and transparent reporting of the data, we followed the guidelines provided by the Strengthening the Reporting of Observational Studies in Epidemiology (STROBE) [6] and Strengthening the Reporting of Cohort, Cross-Sectional, and Case–Control Studies in Surgery (STROCSS) 2021 [7] (Supplementary material).

## Results

A total of 111 patients were included in this study, with 222 specimens sent for histopathological assessment. Results were calculated with number of patients as denominator. Patient demographics are summarised in Table 1. All patients met the AAOHNS 2011 criteria for tonsillectomy, with 92 patients (82.88%) in group A, 12 patients (10.81%) in group B, and 7 patients in group C. Of the 111 patients, 57 (51.35%) were males and 54 (48.64%) were females. Asymmetrical tonsillar enlargement was observed in 8 patients (7.20%).

**Table 1:** Demographic data of study population

Parameter	Frequency	Percentage
*Age (years)*		
Group A(5–18)	92	82.88%
Group B(19–40)	12	10.81%
Group C(> 40)	7	6.30%
*Sex*		
Male	57	51.35%
Female	54	48.64%
*Indications for tonsillectomy (AAOHNS 2011) (Primary)*		
Chronic tonsillitis	105	94.59%
Sleep disordered breathing	2	1.80%
Referred otalgia	4	3.6%
*Symmetry of tonsillar enlargement*		
Symmetrical	103	92.80%
Asymmetrical	8	7.20%
*Histopathological findings*		
Chronic tonsillitis	102	91.89%
Chronic tonsillitis with predominantly follicular hyperplasia	85/102	76.57%
Chronic tonsillitis with predominantly fibrosis	17/102	15.31%
Surface actinomycetes	2/102	1.9%
Chronic granuloma	3	2.7%
Malignancy	5	4.5%
Squamous cell carcinoma	3	2.7%
Non-Hodgkin’s Lymphoma	2	1.8%
Choristoma	1	0.90%

### Histopathological Findings

**Fig. 1:**
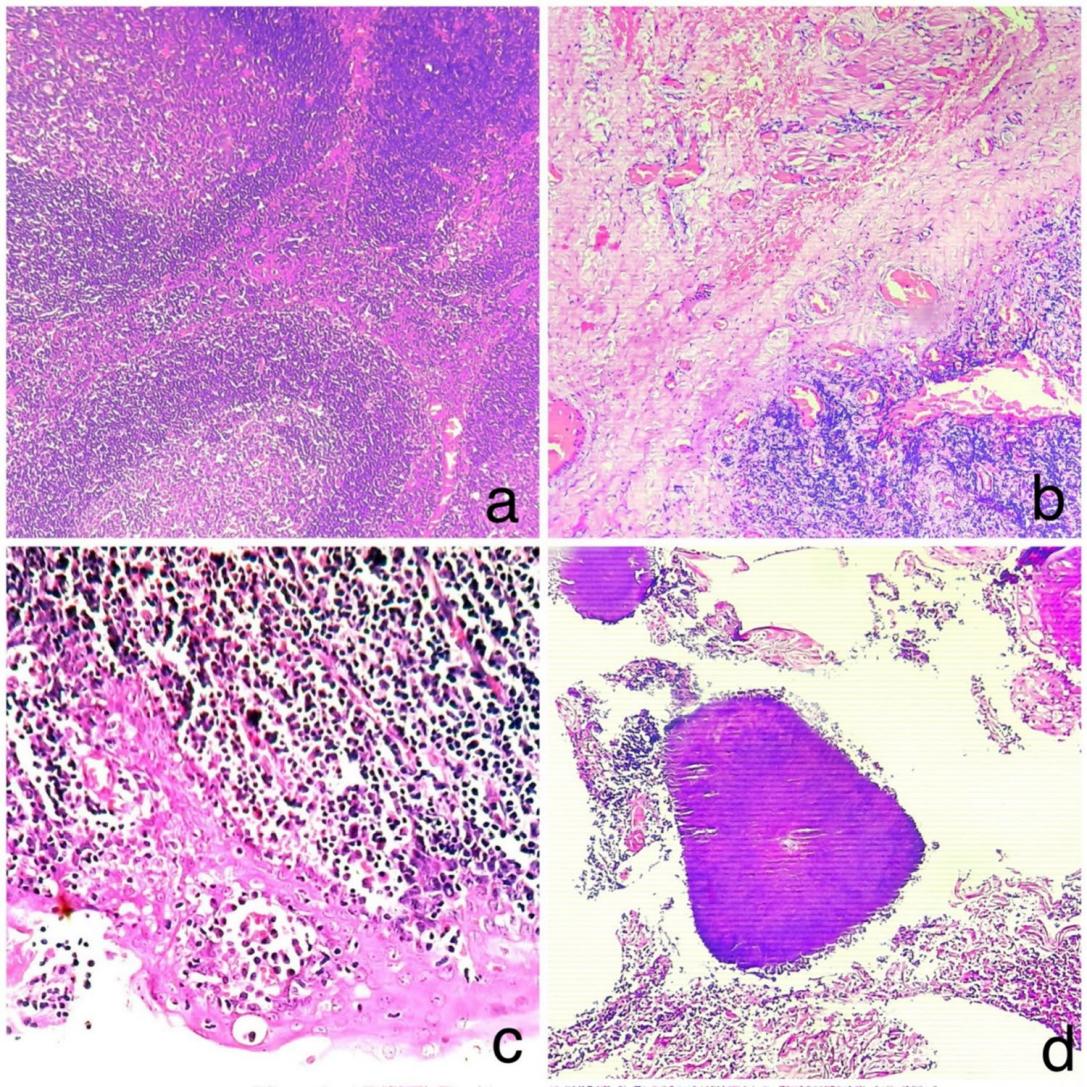
**a** Chronic tonsillitis with follicular hyperplasia, **b** Chronic tonsillitis with fibrosis, **c** Ugras abscess, **d** Colonies of actinomycetes in tonsillar crypts

**Fig. 2:**
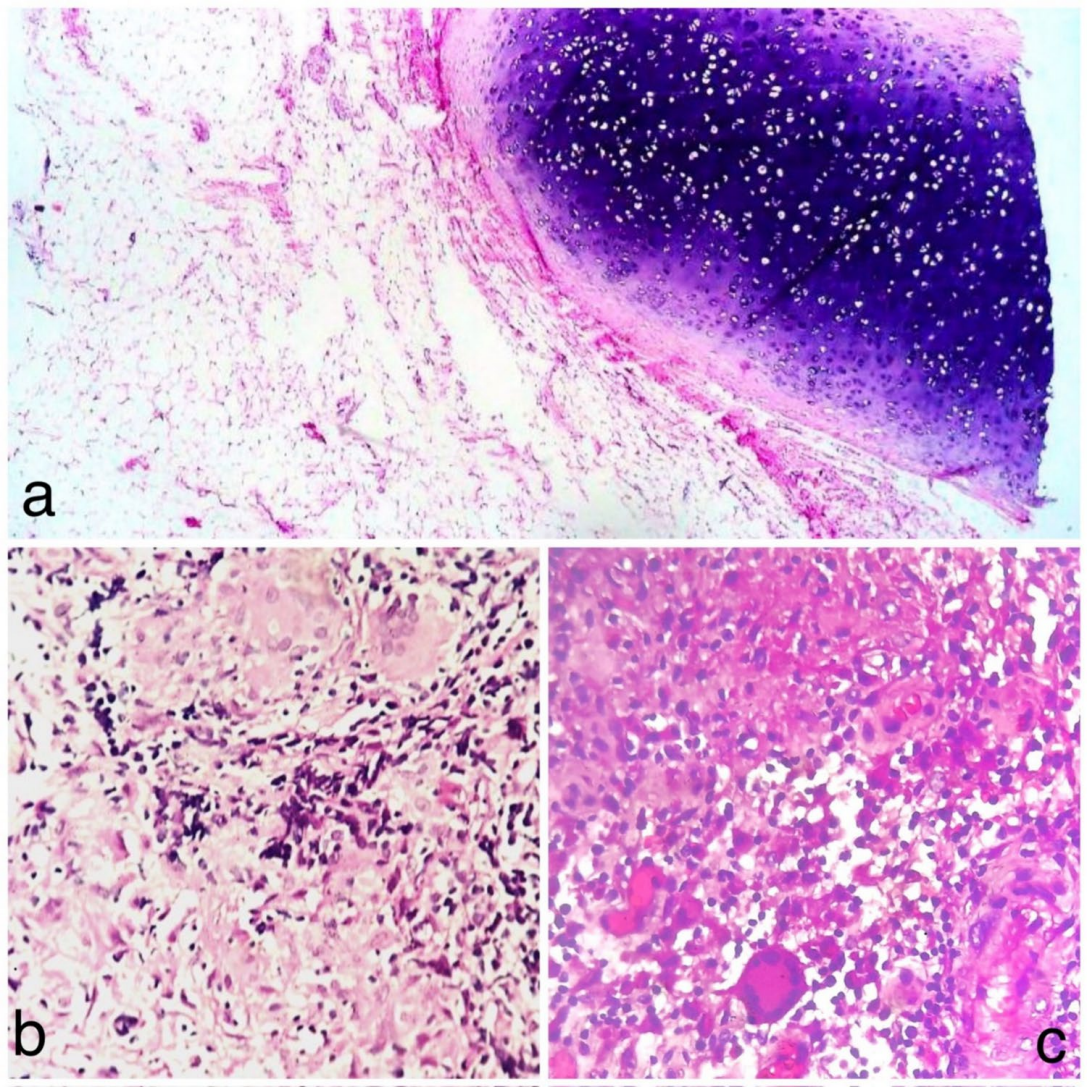
**a** Cartilagenous choristoma in palatine tonsil, **b**, **c** Chronic tonsillar granuloma

**Fig. 3:**
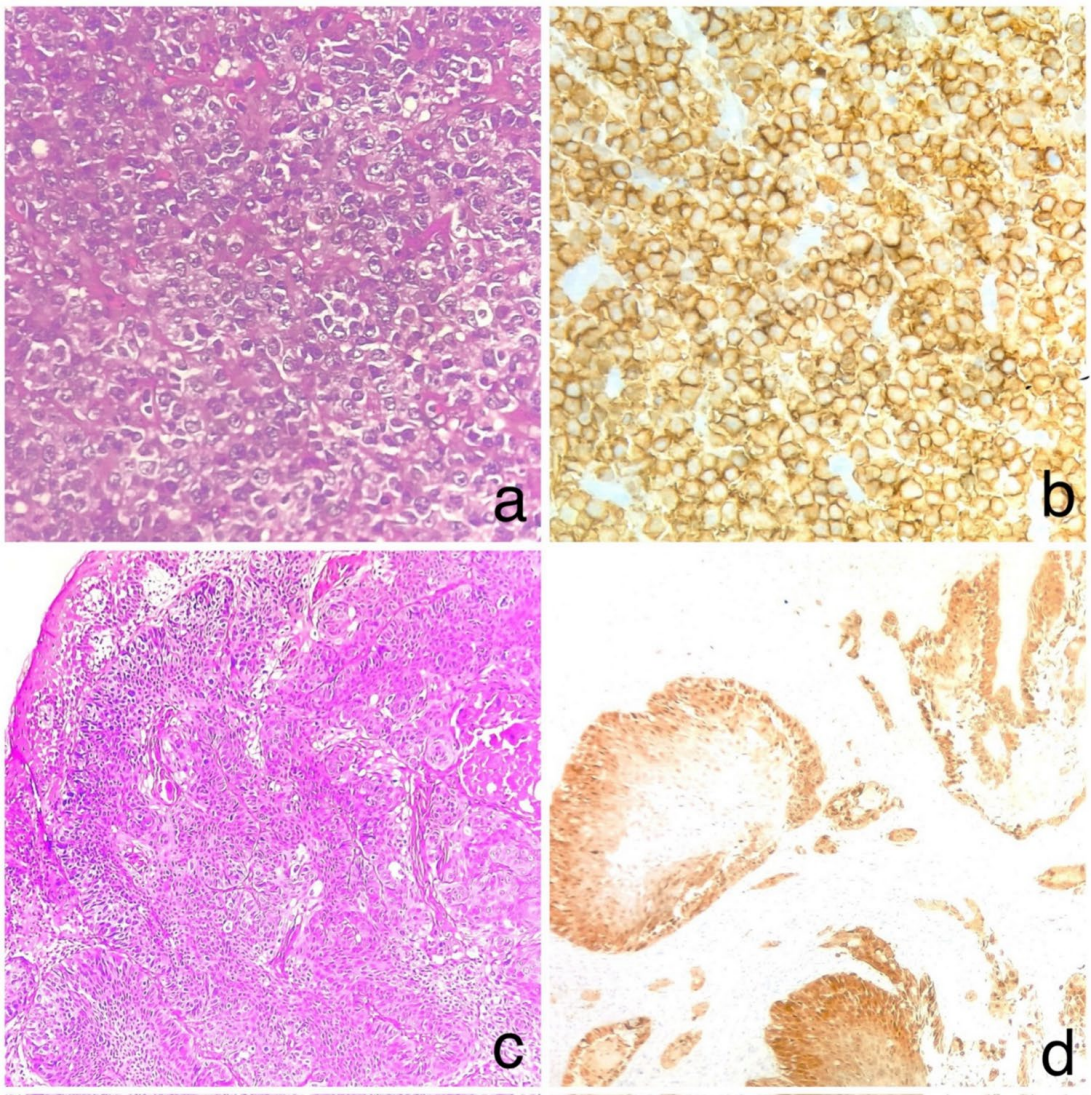
**a** Non-Hodgkin's lymphoma, **b** IHC (CD20), **c** Squamous cell carcinoma, **d** IHC (p16)

A total of 102 patients (91.89%) showed features of chronic tonsillitis on histopathology. Among them, 85 (76.57%) patients exhibited a predominantly follicular hyperplasia pattern and predominantly fibrotic pattern was observed in 17 (15.31%) patients (Fig. 1). Tonsillar chronic granulomas were noted in 3(2.7%) patients (Fig. 2). Squamous cell carcinoma was seen in 3(2.7%), non-Hodgkin's lymphoma were identified in 2 (1.8%) patients (Fig. 3), and cartilaginous choristoma was found in 1 (0.90%) patient (Fig. 2). Quantitative data of histopathological findings are summarised in Table 1**.**

### Correlation of Significant Histopathology with Independent Variables

Tonsillar asymmetry, age more than 18 years and recent onset of symptoms were significantly found to be associated with findings of abnormal histopathology (Table 2).

**Table 2:** Correlation of histopathological findings with independent variables

Parameter	Significant Histopathology (n-9)	Histopathology not significant (n-102)	*p* value	Odds ratio (95% CI)
*Sex*				
Female (0)	3	52	0.48	0.94 (0.84–1.05)
Male (1)	6	50		
*Age*				
Age < 40 (0)	5	100	< 0.0001	3.3 (1.04–10.86)
Age > 40 (1)	4	2		
Age < 18 (0)	4	85	0.002	1.3 (1.02–1.67)
Age > 18 (1)	5	17		
*Tonsillar symmetry*				
Symmetrical (0)	3	100	< 0.0001	3.8 (1.16–12.90)
Asymmetrical (1)	6	2		
*Duration of symptoms*				
> 3 months (0)	5	102	< 0.0001	21.40 (9.09–50.36)
< = 3 months(1)	4	0		

### Correlation of Malignancy with Other Independent Variables

Male sex, presence of asymmetry, age more than 40 and recent onset of symptoms (< = 3 months) were found to be significantly associated with malignancy (Table 3).

**Table 3:** Correlation of malignancy with independent variables

Parameter	Malignancy present (n-5)	No malignancy (n-106)	*p* value	Odd’s ratio (95% CI)
*Age*				
Age < 18 (0)	0	88	< 0.0001	1.2 (1.03–1.58)
Age > 18 (1)	5	18		
Age < 40 (0)	0	104	< 0.0001	3.5 (1.08–11.29)
Age > 40 (1)	5	2		
*Sex*				
Female (0)	0	55	0.008	2.07 (1.70–2.53)
Male (1)	5	51		
*Tonsillar symmetry*				
Symmetry (0)	1	102	< 0.0001	1.9 (0.99–3.96)
Asymmetry (1)	4	4		
*Duration of symptoms*				
> 3 months (0)	0	106	< 0.0001	107 (15.21–752.65)
< = 3 months (1)	5	0		

### Significant Histopathology and Further Management

Based on histopathological findings, out of 9 patients with abnormal histopathology, 8 (7.20%) patients were subjected to further investigations with or without further treatment. Choristoma did not warrant further investigations.

## Discussion

Age categorisation was determined by considering the individuals have an equivocal chance of presenting with both inflammatory and neoplastic lesions in 18–40 years age group [8]. Furthermore, as age progresses beyond 40 years, the likelihood of encountering neoplastic lesions in palatine tonsils increases significantly [8, 9].

Predominant histopathological finding was chronic tonsillitis in 91.89% patients, which justify selection criteria of patients. Predominant pattern of chronic tonsillitis was follicular hyperplasia and less commonly fibrotic pattern. Follicular hyperplasia pattern was predominant in 5–18 age group and fibrotic pattern was predominantly seen in age > 18 years. High immunological activity of palatine tonsils in the paediatric age group and natural involution of palatine tonsils during early adolescence explains these findings [10, 11]. Different percentage of chronic tonsillitis have been reported in literature with majority histopathology showing chronic tonsillitis with variable follicular hyperplasia and fibrosis [12–17]. The results of our research align with previous studies.

Interesting finding in chronic tonsillitis cases was presence of surface actinomycetes in 2 cases. In both cases actinomyces colonies were present in tonsillar crypts without tissue reaction. We did not include actinomycosis as abnormal findings as they are commensals present in oral cavity. Actinomyces are gram positive, filamentous, anaerobic non-acid fast bacteria and are known to colonise tonsillar crypts [18, 19]. Prevalence of actinomycosis in crypts of tonsils vary from 0.8 to 61.6% [18]. Study by Zieba et al. [18] intended to find out clinical significance of actinomycosis in the tonsillectomy specimen and concluded that actinomycetes in tonsil is merely saprophytic microflora and apart from halitosis, there is no significant pathological correlation. Surface actinomycetes without parenchymal tissue reaction are considered as commensal colonies [19–21]. Actinomyces residing deep in crypts may not be amenable to surface tonsillar cultures and symptoms of chronic tonsillitis may not respond to antibiotic therapy and these patients get significant symptomatic relief with tonsillectomy [22]. According to the findings of our study, patients diagnosed with actinomyces showed no other notable clinical features except for chronic tonsillitis and grade 4 tonsillar enlargement, which are in accordance with the literature.

Presence of malignancy in tonsillectomy specimen is the most significant finding. In our study 5 (4.5%) patients were diagnosed to have malignancy based on histopathology and all patients belonged to age group > 40 years. None of the patients had visible lesion on tonsils or suspicious lymphadenopathy which warrants additional imaging before proceeding for diagnostic tonsillectomy. Recent onset of symptoms like foreign body sensation in throat and otalgia, tobacco addiction, asymmetrical tonsillar enlargement were found to be significantly associated with squamous cell carcinoma of tonsil. However both patients diagnosed with non-Hodgkin’s lymphoma did not have any symptoms suspicious of malignancy except recent onset sleep disordered breathing and age more than 40 years. In these patients routine histopathology helped in identifying malignancy and guiding further management.

In 2 patients with squamous cell carcinoma, lesion was localised well within tonsil without breach of tonsillar hemicapsule with all margins minimum 5 mm away from the lesion. Post-surgery imaging did not reveal residual lesion or suspicious lymphadenopathy. Both patients denied prophylactic neck treatment in the form of neck dissection or irradiation and opted for 3 monthly follow up with neck ultrasonography and cross sectional imaging as and when required. One patient had close but free base i.e. 0.2 cm and thus received adjuvant chemoradiation to primary and neck. Two patients with non-Hodgkins lymphoma underwent further imaging for staging and turned out to be localised disease in tonsils. Both patients received definitive chemotherapy in the form of CHOP regimen. In all 5 patients routine histopathology helped in identifying malignancy in early stage and could receive curative treatment well in time.

In a systematic review by Rokkjaer et al. [23] incidence of unsuspected malignancy was 0.015%, without presence of risk factors such as asymmetry of tonsil, visible lesion, cervical lymphadenopathy, previous malignancy, history of radiotherapy, constitutional symptoms, weight loss or immunodeficiency. They concluded that rarity of presence of malignancy in unsuspected patients doesn’t justify routine histopathology. Systematic review by Randall et al. [13] gave 0.087% prevalence of malignancy with 0.011% prevalence of true occult malignancies with a conclusion that routine histopathology is not warranted in absence of suspicion. Individuals studies reporting routine histopathology and consensus based on study have been summarised in Table 4.

**Table 4:** Studies reporting routine histopathology of tonsillectomy specimen and malignancy in tonsillectomy specimens

References	Year	Study design	Sample size	Age group	Malignancy	Consensus of the study
Starry et al. [24]	1939	Not specified	8538	Not specified	2 cases	Recommended routine HPE of tonsils
Weibel et al [9]	1964	Retrospective	3627 (only tonsillectomy specimens)	3 months-70 yr	None had unsuspected malignancy	Routine HPE should be restricted to patients older than 40 years
Dohar et al. [25]	1996	Survey and retrospective analysis	27	Paediatric	1 Lymphoma	No definite consensus, HPE is based on institutional protocol or medicolegal cover
Alvi et al. [26]	1998	Retrospective	288 (576 specimens)	1–68 yr	1 Lymphoma	Routine HPE is not necessary, should be done in patients with asymmetric tonsils and history of malignancy
Beaty et al. [27]	1998	Retrospective	476	> = 18 yr	25	All 25 patients had clinical risk factors. Risk model predictive of malignancy can be constructed
Ikram et al. [28]	2000	Retrospective	200	4–49 yr	1 NHL	Routine HPE is not necessary in absence of pre-operative risk factors
Erdag et al. [29]	2005	Retrospective	1930	< 19 yr	None	Routine HPE is not necessary in paediatric tonsillectomy in absence of preoperative risk factors
Mohamad et al. [17]	2007	Retrospective	241	2–64 yr	None	Routine HPE is not necessary in paediatric age group
Agoda et al.[30]	2011	Retrospective	61	1–50	1 ( Lymphoma)	HPE request should be based on risk factors
Rebechi et al.[1]	2013	Retrospective	281	2–22	None	Routine HPE is dispensable and increases cost of surgery
Aksakal et al. [14]	2018	Retrospective	1356 (only tonsillectomy)	1–81 yr	2 (0.1%)—lymphoma	Routine HPE is necessary in all cases of adult tonsillectomy. Risk factors to be considered in paediatric population
Manzoor et al. [31]	2018	Cross sectional	132	5–69 yr	1 Squamous cell carcinoma 1 NHL	HP plays important role in patients presenting with chronic tonsillitis in reducing associated morbidity
Sharma et al. [15]	2020	Retrospective	105	1–70 yr	2 cases of Squamous cell carcinoma 1 case of Lymphoma	HPE plays significant role in diagnosing both benign and malignancy conditions
Nikethan et al. [32]	2020	Cross sectional	156	5–60 yr	1 squamous cell carcinoma	Routine HPE has low cost- benefit rate and diagnostic tool for malignancy
Varute et al. [16]	2022	Prospective	200	5–39 yr	None	No need for HPE in absence of associated risk factors
Kandemir et al. [33]	2023	Retrospective	635	3–48 yr	No malignancy or unexpected findings	HPE request should be based on specific risk factors
Modh et al. [4]	2023	Observational	35	3–37 yr	Nil	Routine HPE is recommended to know the cause of enlarged tonsil

Presence of tonsillar lesion, suspicious hard cervical lymphadenopathy and previous history of head and neck malignancy; warrants further imaging before proceeding with diagnostic tonsillectomy. In such cases biopsy from lesion or FNAC from cervical lymph node will be needed to establish diagnosis of malignancy and diagnostic tonsillectomy is deferred as its oncologically not safe. Tonsillectomy performed in adult population is usually a diagnostic tonsillectomy to rule out malignancy. It is prudent to say that routine histopathology can diagnose malignancy in grossly normal looking tonsil than merely calling them as clinically unsuspected cases.

Our study suggests that there is a small but definite risk of malignancy in absence of visible tonsillar lesion or suspicious lymphadenopathy. Early detection and prompt treatment of early malignancy can be life saving for the patient and outweighs cost of routine histopathology.

In this study, 2.7% of the participants were found to have chronic granulomas (Fig. 2b, c) on histopathology. Chronic granulomas were seen in patients under 40 years with symptoms of recurrent tonsillitis. In order to assess the possibility of systemic granulomatous disease, particularly tuberculosis given the endemic nature of the disease in India, all three patients underwent additional evaluation. One patient was found to have pulmonary tuberculosis, another had tuberculous lymphadenitis, while the third patient did not exhibit any signs of systemic granulomatous disease. The histopathological examination played an important role in identifying active tuberculosis and subsequent treatment.

Primary tonsillar tuberculosis is rare due to antiseptic action of saliva, inherent immunological activity of tonsil, thick stratified epithelium and presence of commensals interfering pathogenic colonisation [34, 35]. Tonsillar tuberculosis can be primary i.e. in absence of pulmonary tuberculosis and secondary i.e. co-existing with pulmonary tuberculosis. Isolated cases of primary tonsillar tuberculosis diagnosed on histopathology have been reported [34–39]. According to Kardon et al. [40] presence of granuloma in tonsil is suggestive of systemic disease which warrants further evaluation. In their retrospective study of 22 cases based on histochemical and clinical follow-up information, the aetiology of the granulomas included sarcoidosis (n = 8), tuberculosis (n = 3), Hodgkin's lymphoma (n = 2), toxoplasmosis (n = 1), squamous cell carcinoma (n = 1), and no specific known cause (n = 7). They concluded that aetiology may not be identifiable in each case but careful workup is necessary to avoid clinical misdiagnosis. In 23 cases of adenotonsillar granuloma Al-Sebeih et al. [41] found only 1 case of tuberculosis and concluded that routine histopathology is doubtful. However in endemic areas like India tuberculosis remains first suspicious systemic granulomatous disease [35]. Tonsillar tuberculosis may be representative of systemic or pulmonary tuberculosis, which warrants anti tubercular treatment. As per our study routine histopathology of resected tonsils is justified owing to potential serious complications associated with untreated cases of tuberculosis.

Choristoma is abnormal location of normal tissue [42]. Choristoma was first described by Berry in 1890 [43]. Tonsillar choristoma commonly presents as painless tonsillar enlargement or chronic tonsillitis [43, 44]. Tonsillectomy remains curative and gives symptomatic relief to patient. Cartilaginous choristoma is usually incidental finding after routine histopathology. Multiple case reports of cartilaginous choristoma in tonsil after histopathology have been reported in literature [43–47]. In the prospective analysis by Ciris et al. [44], tonsils of 141 patients out of 2355 were found to have cartilage choristoma, out of which 75.70% patients were operated for recurrent tonsillitis and 24.29% patients had idiopathic tonsillar hypertrophy. In accordance with the literature, one patient in our study had asymmetrical tonsillar enlargement with symptoms of recurrent tonsillitis was found to have cartilaginous choristoma in enlarged tonsil (Fig. 2a). Referred otalgia was additional complaint of the patient. Patient had significant symptomatic relief post-surgery.

The allocation of pathologist working hours is a finite resource. Routine histopathological examination of tonsillectomy specimens can increase the workload of pathologists, potentially leading to backlogs and delays in reporting results for more urgent cases. However tonsillectomy specimen is a small specimen requiring 2 to 4 sections. Overall time required for tissue processing and examination is relatively less due to small size of specimens.

Routine histopathological examination of tonsillectomy specimens incurs additional costs for patients. However, considering cost benefit ratio of early detection of malignancy or tuberculosis, cost of routine histopathology can be justified. Understanding the economic implications of routine histopathological examination is vital for healthcare policymakers, as it allows for the assessment of cost-effectiveness and allocation of resources towards more clinically significant investigations. In a previous study conducted by Chinawa et al. [48], it was observed that the cost of routine adeno-tonsillar histopathology was 26 US dollars. However, they did not find any significant histopathological findings. As a result, they concluded that routine histopathology may not be necessary due to a negative cost benefit ratio. In contrast to the study mentioned, our research revealed a notable occurrence of significant histopathological findings in routine tonsillectomy specimens that appeared normal upon visual examination. These findings necessitated additional treatment and intervention. Given the potential health implications of undiagnosed malignancies and tuberculosis, the cost of routine tonsillectomy can be justified.

The study's strength lies in its prospective design, enabling real-time data collection from consecutive patients. Patients were selected according to the standard guidelines set by the AAOHNS-2011. Conducted in a general hospital, the study provides a representative sample of the general population. However, it is important to note that the study was conducted in a government hospital setting, where treatment provided is free of cost to below poverty line patients and at a nominal cost to others. Therefore, the cost benefit analysis may not directly apply to private or semi-government hospital settings due to potential variations in treatment costs.

## Conclusions

In order to assess the prevalence of significant tonsillar diseases that warrant further management, we conducted a Prospective study on the benefits of routine histopathology in tonsillectomy specimens. We found that there is a low but definitive risk of malignancy, such as non-Hodgkin’s lymphoma and early squamous cell carcinoma, in unsuspected tonsillectomy specimens. Considering the benefits of early diagnosis and treatment of early malignancy, systematic granulomatous diseases, and the relatively uncomplicated nature of the procedure for pathologists, routine histopathology should be considered after tonsillectomy.

When cost constraints are a concern, risk-based approach can be adopted. Factors such as age (more than 18 years), asymmetry of tonsils, referred otalgia, recent onset and short duration of symptoms and a history of addiction should be taken into consideration as significant indicators to proceed with histopathology. By carefully weighing the potential benefits and costs, healthcare providers can make informed decisions regarding routine histopathological examination in tonsillectomy cases.

## Supplementary Information

Below is the link to the electronic supplementary material.

Supplementary file1 (DOCX 160 kb)
